# Novel point mutations attenuate autotaxin activity

**DOI:** 10.1186/1476-511X-8-4

**Published:** 2009-02-17

**Authors:** Eunjin Koh, Russell W Bandle, David D Roberts, Mary L Stracke, Timothy Clair

**Affiliations:** 1 University of Virginia School of Medicine, Department of Cell Biology, Charlottesville, Virginia 22908, USA; 2 Laboratory of Pathology, Center for Cancer Research, National Cancer Institute, National Institutes of Health, Bethesda, MD 20892-1500, USA

## Abstract

**Background:**

The secreted enzyme autotaxin (ATX) stimulates tumor cell migration, tumorigenesis, angiogenesis, and metastasis. ATX hydrolyzes nucleotides, but its hydrolysis of lysophospholipids to produce lysophosphatidic acid (LPA) accounts for its biological activities. ATX has been identified only as a constitutively active enzyme, and regulation of its activity is largely unexplored. In spite of its presence in plasma along with abundant putative substrate LPC, the product LPA is found in plasma at unexpectedly low concentrations. It is plausible that the LPA-producing activity of ATX is regulated by its expression and by access to substrate(s). For this reason studying the interaction of enzyme with substrate is paramount to understanding the regulation of LPA production.

**Results:**

In this study we determine ATX hydrolytic activities toward several artificial and natural substrates. Two novel point mutations near the enzyme active site (H226Q and H434Q) confer attenuated activity toward all substrates tested. The Vmax for LPC compounds depends upon chain length and saturation; but this order does not differ among wild type and mutants. However the mutant forms show disproportionately low activity toward two artificial substrates, pNpTMP and FS-3. The mutant forms did not significantly stimulate migration responses at concentrations that produced a maximum response for WT-ATX, but this defect could be rescued by inclusion of exogenous LPC.

**Conclusion:**

H226Q-ATX and H434Q-ATX are the first point mutations of ATX/NPP2 demonstrated to differentially impair substrate hydrolysis, with hydrolysis of artificial substrates being disproportionately lower than that of LPC. This implies that H226 and H434 are important for substrate interaction. Assays that rely on hydrolyses of artificial substrates (FS-3 and pNpTMP), or that rely on hydrolysis of cell-derived substrate, might fail to detect certain mutated forms of ATX that are nonetheless capable of producing LPA in the presence of sufficient exogenous substrate. H420Q-ATX could not be differentiated from WT-ATX, indicating that histidine at position 420 is not required for any of the activities of ATX tested in this study.

## Background

Autotaxin (ATX, NPP2), a secreted enzyme originally purified as a tumor cell motility-stimulating factor [1], enhances the tumorigenic and metastatic potential of transformed cells [2], and stimulates angiogenic activity in Matrigel™ plug assays [3], thereby affecting several facets of oncogenic progression. ATX expression has been implicated in development [4-6], and lymphocyte trafficking [7], and has been associated with a number of pathologies including adipose tissue metabolism [8], rheumatoid arthritis [9], Alzheimer type dementia [10], multiple sclerosis [11], neuropathic pain [12], and a growing list of cancer types [13].

Although products of the ATX nucleotide phosphodiesterase activity, such as adenosine nucleotides [14], have been found to possess motility-stimulating activity [15], adenosine analogs that blocked this A1-receptor-mediated activity had no effect on ATX-stimulated motility indicating that nucleotide products were not the mediators of ATX-stimulated migration [16].

The biological activities of ATX are now understood to arise from its lysophospholipase D (LPLD) activity [13] that catalyzes the conversion of lysophosphatidylcholine (LPC) and other lysoglycerophospholipids to lysophosphatidic acid (LPA). A number of point mutations, each of which abolished both the LPLD and nucleotide phosphodiesterase (nucleotide PDE) activities of ATX, were found to eliminate ATX-stimulated motility. Hence, the enzymatic active site for nucleotide PDE and LPLD coincide and this intact site is required for ATX to act as a motogen [16,17].

The LPLD, nucleotide PDE, and motility-stimulating activities of ATX all require the presence of threonine at (human sequence) position 210 (T210) as well as three metal-binding histidines (H316, H360, H475) [16,17]. The catalytic domain, as well as the C-terminal and N-terminal domains, are necessary for ATX's unique LPLD activity; and a point mutation within the catalytic domain of murine ATX (L211H equivalent to L214 in human ATX) enhanced the nucleotide PDE activity while decreasing the LPLD activity [18]. To our knowledge, this is the only published report of a point mutation in ATX that affects substrate preference.

We have identified two novel point mutations in the vicinity of the active site of ATX that disproportionately discriminate against the hydrolysis of two artificial substrates (FS-3 and PpNpTMP), have attenuated activity toward LPC, and are defective in the stimulation of migration. In this manuscript, we define the Km and Vmax of native ATX for its various substrates, and characterize the altered kinetic and biological properties of ATX-H226Q and ATX-H434Q.

## Results and discussion

### Expression of novel mutants

**Figure 1:**
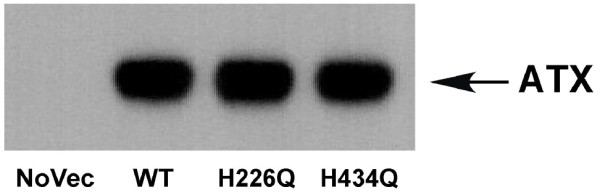
**Immunoblot of autotaxin preparations**.

Nucleotide pyrophosphatase and phosphodiesterases (NPPs) are metallo-enzymes that contain a number of histidine and aspartate residues in the presumed metal-binding site. Several of these residues have been shown to be required for their enzymatic activities [16,17,19]. We used site-directed mutagenesis to introduce novel histidine-to-glutamine alterations (H226Q, H420Q, and H434Q) in the vicinity of this NPP active site in ATX and harvested conditioned media from transiently transfected COS-1 cells to obtain these mutant forms of ATX. Fig. 1A shows an immunoblot of concentrated conditioned media containing no ATX (NoVec), wild type (WT-ATX), H226Q-ATX, or H434Q-ATX at normalized protein concentrations.

### H226Q-ATX and H434Q-ATX are substrate-selective

**Figure 2:**
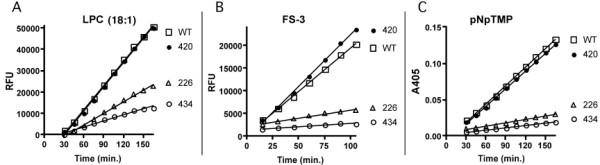
**Substrate selectivity of H226Q and H434Q**. Hydrolyses were detected as described in Methods. Reactions contained 60 ng ATX protein and product accumulation was read beginning at 30 minutes and at intervals thereafter as indicated. A. [LPC] = 125 μM. B. [FS-3] = 6 μM. C. [pNpTMP] = 4 mM. WT: wild type; 420: H420Q; 434: H434Q; 226: H226Q.

The enzymatic activities of the wild type vs. mutant forms of ATX were tested for three structurally distinct types of ATX substrates. Fig. 2 shows a comparison of hydrolytic activities toward LPC (18:1, the putative natural substrate) at 125 mM, toward FS-3 (an auto-quenching fluorescent synthetic analog of LPC) at 6 mM, and toward 4 mM p-nitrophenyl-TMP (pNpTMP, a nucleotide analog). These substrate concentrations are approximately equal to Km values for wild type ATX as determined in preliminary experiments (data not shown). Initial pseudo-first order reaction rates for WT-ATX and H420Q-ATX were indistinguishable for all three substrates. Compared to WT-ATX, H226Q-ATX and H434Q-ATX showed lower but significant hydrolytic activity toward LPC (48 and 26% of WT, respectively). However, both H226Q-ATX and H434Q-ATX showed parallel, but disproportionately lower, activities toward the artificial substrates, FS-3 (19 and 8% of WT, respectively) and pNpTMP (19 and 13% of WT, respectively). Conditioned medium containing T210A-ATX showed no hydrolytic activity toward any of the three substrates tested (data not shown). These data indicate that alteration of H226 or H434 resulted in lowered activity and substrate-selectivity. H226Q and H434Q mutant proteins possessed sufficient activity to hydrolyze LPC (18:1), but could escape detection in FS-3 or pNpTMP hydrolysis assays.

### H226Q and H434Q have altered kinetic parameters

**Figure 3:**
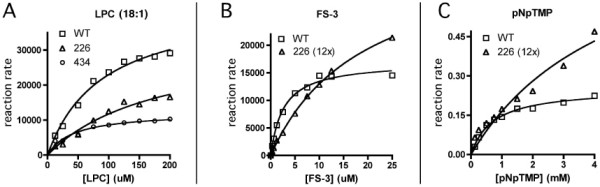
**Kinetic analysis**. Hydrolyses were detected as described in Methods. A. LPC hydrolysis. Reactions contained 30 ng ATX protein. B. FS-3 hydrolysis. Reactions contained 30 ng (WT) or 360 ng (H226Q) ATX protein. C. pNpTMP hydrolysis. Reactions contained 30 ng (WT) or 360 ng (H226Q) ATX protein. For all reactions, product formation was linear (R^2 ^= 0.99) between 30 minutes and 3 hours. Data are expressed as relative reaction rates [RFU (or A405)/time] and plotted against substrate concentrations. WT: wild type; 420: H420Q; 434: H434Q; 226: H226Q.

To better understand the altered hydrolytic efficiency and substrate selectivity of H226Q-ATX and H434Q-ATX, we determined the effects of substrate concentrations on the reaction rates and calculated the kinetic parameters Km and Vmax for each substrate. Fig. 3A shows the relative hydrolytic activities of WT-, H226Q-, and H434Q-ATX (each at 30 ng ATX protein/reaction) as a function of LPC (18:1) concentration. Because of the low activity of H226Q-ATX toward the substrates FS-3 and pNpTMP, we increased the concentration of this mutant ATX to 12 times that of WT-ATX. Fig. 3B shows the relative hydrolytic activities of WT-ATX (30 ng ATX protein/reaction) and H226Q-ATX (360 ng ATX protein/reaction) as functions of FS-3 concentration, and Fig. 3C shows the relative hydrolytic activities of WT-ATX and H226Q-ATX (protein/reaction as in B) as functions of pNpTMP concentration.

Km and Vmax values from these data, normalized for ATX protein concentration, are included in Table 1 along with additional data described in the following section. When H226Q-ATX is compared to WT-ATX, Vmax is lower for all substrates tested, but these changes are disproportionately large for FS-3 and pNpTMP compared to LPC (18:1). These data explain the substrate selectivity noted in initial rates of digestion. Data values for hydrolysis of FS-3 and pNpTMP by H434Q-ATX were too low, and contained too much scatter, to yield reliable values for kinetic parameters (data not shown).

**Table 1:** Kinetic Parameters

		**Vmax**		
	**WT**	**H226Q**	**H434Q**	**H420Q**

LPC 16:0 ^a^	306 ± 60	149 ± 63	111 ± 26	359 ± 63

LPC 18:0 ^a^	129 ± 56	76 ± 37	NS	149 ± 49

LPC 18:1 ^a^	589 ± 96	267 ± 59 *	107 ± 13 *	472 ± 143

FS-3 ^a^	142 ± 4	26 ± 1	NS	ND

pNpTMP ^b^	21 ± 0.8	7 ± 2.0	NS	ND

		**Km**		

	**WT**	**H226Q**	**H434Q**	**H420Q**

LPC 16:0 ^c^	66 ± 11	126 ± 9 *	120 ± 15 *	94 ± 8

LPC 18:0 ^c^	89 ± 9	270 ± 152	NS	115 ± 31

LPC 18:1 ^c^	73 ± 8	126 ± 20	105 ± 21	69 ± 18

FS-3 ^c^	2.6 ± 0.3	19 ± 1.1	NS	ND

pNpTMP ^d^	0.7 ± 0.1	4.8 ± 2.2	NS	ND

^a^: Units are RFU/min./μg ATX protein ± SEM.

^b^: Units are A405 × 10000/min./μg ATX protein ± SEM.

^c^: Units are μM ± SEM, ^d^: Units are mM ± SEM.

*: p < .05 vs WT (one-way ANOVA with Dunnett's post test).

NS: data not significant, ND: not determined.

### Effects of acyl chain length and saturation

We compared the kinetic parameters of the mutant forms toward LPC 16:0, LPC 18:0, and LPC 18:1; these results are also included in Table 1. For each species of LPC, the mutant's activity, as assessed by determination of Vmax, was attenuated compared to wild type; but the order of activity profile (18:1 > 16:0 > 18:0) did not differ from wild type, and was consonant with previous reports [20,21].

The kinetic data demonstrate the strengths and weaknesses of each enzymatic assay for the detection of ATX activity. Product formation kinetics were linear at 30 – 100 ng WT-ATX protein for FS-3 and LPC, and at 30 – 60 ng WT-ATX protein for pNpTMP (data not shown). LPC has the advantage of being ATX-specific. Although FS-3 hydrolysis by NPP1 and NPP3 is considered unlikely because they lack activity toward lysophospholipids, direct evidence that FS-3 itself is resistant to hydrolysis by NPP1 or NPP3 has not been reported and this limits the interpretation of ATX specificity [22]. In contrast, pNpTMP is well known to be susceptible to hydrolysis by other NPPs. LPC has the additional advantage of being a naturally occurring lysophospholipid whose presence can be confirmed in a biological assay. A limitation of the LPC enzymatic assay is that detection is coupled to choline oxidase/peroxidase, which is itself susceptible to inhibition, depending on the conditions of the assay. In addition, Amplex^® ^Red, the fluorescent detector for choline oxidase activity, may be susceptible to direct hydrolysis, which we occasionally observed in partially purified ATX preparations from transfected insect (HiFive) cell conditioned medium (data not shown). Notably, none of the ATX preparations used in this study (from transfected COS1 cell conditioned media) hydrolyzed Amplex^® ^Red directly (data not shown).

### Comparison of Migration Responses

**Figure 4:**
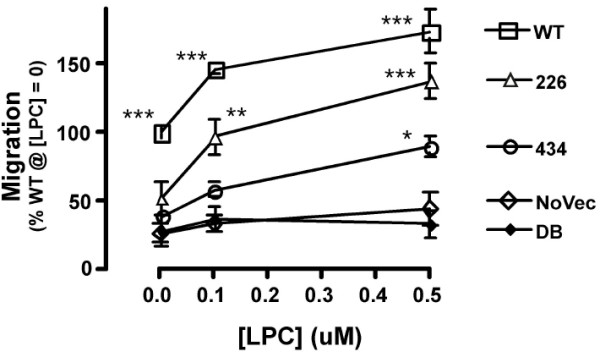
**Effect of LPC on migration responses**. Values expressed as % WT at [LPC] = 0 (100% = 358 ± 24 nuclei/low power field). The total number of nuclei counted in this experiment: 118,472. Data are shown as mean ± SEM, (DB and NoVec: two chambers; others: four chambers) and were compared by two-way ANOVA and Bonferroni's post-test (*: p <.05, **: p <.01, ***: p <.001, each compared to NoVec) utilizing Prism 4.0b (GraphPad Software, Inc, San Diego, CA). WT: wild type; 420: H420Q; 434: H434Q; 226: H226Q.

Migration responses of A2058 cells, with or without inclusion of exogenous LPC (18:1), to WT-, H226Q-, and H434Q-ATXs are shown in Fig. 4. In the absence of exogenous LPC, WT-ATX produced its maximal response at the concentration of ATX used (0.23 μg/ml) (data not shown). At the tested concentrations of LPC (0, 0.1, and 0.5 μM), the migration response to NoVec did not differ from the background response to medium alone. WT-ATX stimulated migration significantly above background in the absence of exogenous LPC (p < 0.001 compared to NoVec), suggesting that WT-ATX can generate LPA from cell-derived substrate. Addition of exogenous LPC further stimulated the response, suggesting that cell-derived substrate can be rate limiting.

Migration stimulation by H420Q-ATX was indistinguishable from WT-ATX (data not shown). At tested concentrations, migration stimulation by H226Q-ATX or by H434Q-ATX, without exogenous LPC, was not significantly different from background. Inclusion of sufficient exogenous LPC with either mutant resulted in a significant migration response. Together these results suggest that, compared to WT-ATX, H226Q-ATX and H434Q-ATX are not only less efficient in hydrolyzing exogenously-provided LPC but are also less efficient in hydrolyzing and/or retrieving cell-derived substrate. LPC alone failed to stimulate migration responses in the absence of exogenous ATX. Therefore the migration response data reflect properties intrinsic to the various ATX constructs.

**Figure 5:**
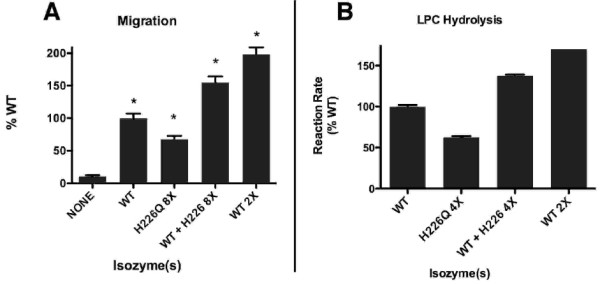
**H226Q does not inhibit the wild type migration response or hydrolytic activity**. A. Migration: Values expressed as in Fig. 4. [WT-ATX] = 0.06 μg/ml; [H226Q-ATX] = 0.46 μg/ml, as indicated. *: p < .001 compared to no attractant. B. LPC Hydrolysis: Values (mean ± SEM) expressed as % WT-ATX. [WT-ATX] = 10 ng/reaction, [H226Q-ATX] = 40 ng/reaction, [LPC, 18:1] = 100 μM.

The product of extra-cellular ATX activity is LPA, a powerful stimulatory lipid which acts via cell surface receptors and whose concentration must be tightly regulated in space and time. This suggests that ATX itself is localized near its products' site of action. If both WT-ATX and the attenuated mutants, e.g. H226Q-ATX, have a common target, competition could occur between the two forms. To detect a dominant negative effect of H226Q-ATX on WT-ATX activity, we tested the migration response of WT-ATX in the presence and absence of excessive amounts of H226Q-ATX, and the results are shown in Fig. 5A.

At higher concentrations of H226Q-ATX (0.46 μg/ml in Fig. 5A compared to 0.23 μg/ml in Fig. 4), there was significant stimulation of migration in the absence of exogenous LPC. At 0.46 μg/ml this activity of H226Q-ATX contributed additively to the total activity when combined with WT-ATX (Fig. 5A). We therefore did not detect inhibition of the migration response to WT-ATX by H226Q-ATX at 8-fold excess.

We also measured the effect of excess H226Q-ATX on the enzymatic (LPC hydrolysis) activity of WT-ATX, and the results are shown in Fig. 5B. Both WT-ATX and H226Q-ATX alone showed significant enzymatic activity and, in combination, these activities were approximately additive. We therefore did not detect inhibition of WT-ATX LPC hydrolysis by H226Q-ATX at 4-fold excess.

The prevailing context of this study was cancer theory and therapy, but as noted in the Background section, the findings have implications for development and for other pathologies. ATX is extra-cellular and a metallo-enzyme; therefore it is an attractive pharmacological target. Knowledge of the details of the lysophospholipase D enzyme mechanism can inform efforts at drug design. For example, ATX and the other NPPs use a "ping-pong" type mechanism that involves two hydrolytic steps. The second step is an attack by water on the covalent enzyme intermediate, and this releases the final product, LPA. Enzymes using this type of mechanism are often sensitive to product inhibition, and it is now apparent that the proposed natural inhibitor, S1P, and the recently reported synthetic inhibitors, are either a product or product analog.

The role of particular amino acids within ATX in this enzyme mechanism is incompletely understood. Studies using chimeric constructs with regions from other NPPs indicate that regions throughout the entire length of ATX are irreplaceable for LPC hydrolysis. It is known that threonine at position 210, the site of intermediate formation, is absolutely required; and certain histidine residues have been implicated as coordinators of required metals. Histidine residues within enzymes can also participate directly in other aspects of catalysis. For example, in histidine phosphatase, histidine plays a crucial role in the phospho-transfer reaction [23]. Measuring LPC hydrolysis and cell migration, we screened a panel of ATX mutants lacking particular histidine residues near to the region of histidine residues already known to be required for activity.

Two mutants, H226Q-ATX and H434Q-ATX showed anomalous results: they could hydrolyze LPC, albeit at a reduced rate, but did not stimulate cell migration. The initial study, identifying plasma LPLD as ATX [24] reported that addition of exogenous LPC stimulated the migration response to low, but not high, concentrations of ATX. This indicated that cell-derived substrate could be rate limiting, and that sufficient concentrations of ATX could overcome this limitation. Indeed in this present study, addition of exogenous LPC supported significant migration responses to the anomalous mutants, confirming that cell-derived substrate was rate-limiting, and disproportionately so for H226Q-ATX and H434Q-ATX. The discovery of these attenuated mutants supports the conclusion that the local production of LPA involves not only catalysis, but also retrieval of endogenous substrate.

## Conclusion

H226Q-ATX and H434Q-ATX are the first point mutations of ATX/NPP2 demonstrated to differentially impair substrate hydrolysis, including that of endogenous substrate(s) in the migration assay. These two histidine residues appear to be important in ATX-substrate interaction. H420Q-ATX could not be differentiated from WT-ATX, indicating that histidine at position 420 is not required for any of the activities of ATX tested in this study. There was no apparent cross-inhibition of either migration stimulation or hydrolytic activity between WT-ATX and the attenuated mutant H226Q-ATX.

## Methods

### Materials

Except as noted, reagents were from Sigma-Aldrich (St. Louis, MO). FS-3 was from Echelon Biosciences (Salt Lake City, Utah), and Amplex UltraRed was from Invitrogen Molecular Probes (Eugene, OR).

### Preparation and detection of Autotaxin Proteins

The HA-tagged ATX was obtained by amplification utilizing the Platinum Pfx DNA polymerase (Invitrogen Life Technologies, Carlsbad, CA). An antisense oligonucleotide primer was used to introduce the HA epitope tag (YPYDVPDYA) and an XbaI site into the C terminus of ATX:

(5'ATGCATGCTCTAGAATAGTCAGGAACATCGTATGGGTACTCGAGAATCTCGCTCTCATATGT3'). The sense primer that we utilized to introduce a HindIII site was (5'ATCTATCTAAGCTTATGGCAAGGAGGAGCTCGTT3'). ATX/NPP2 cDNA, derived from MDA 435 cells cloned into pcDNA3.1 vector (pcDNA3.1wATX/NPP2) was used as a template [2]. The PCR products were digested with HindIII and XbaI and subsequently ligated into pcDNA3.1 (+) to produce pcDNA3.1/HA-ATX

All point mutants of ATX were constructed utilizing the QuikChange XL Site-Directed Mutagenesis Kit from Stratagene per the manufacturer's protocol (La Jolla, CA). Each mutant plasmid was sequenced to confirm the presence of the mutation and the fidelity of the PCR amplification. COS-1 cells were transiently transfected with pcDNA3.1-wtATX or with each mutant plasmid using Lipofectamine 2000 reagent (Invitrogen Life Technologies) per the manufacturer's protocol. A non-vector control was produced by adding appropriate medium and identical treatments but no vector to COS-1 cells. Following transfection, medium was switched to DMEM and cells were incubated for 48 hr. The conditioned media were collected, concentrated, and analyzed by immunoblot using 4–12% polyacrylamide gels, anti-ATX-peptide rabbit polyclonal affinity-purified primary antibody (Ab84A; 1:10,000 dilution), goat-anti-rabbit HRP-conjugated secondary antibody, ECL-plus (Amersham), and quantification by direct scanning (Image Station 4000 MM, Eastman Kodak Company, Rochester, NY). Relative ATX protein concentrations were determined and normalized prior to the assays by comparing immunoblot signal intensities.

### Migration Assay

Cell migration was measured using A2058 human melanoma cells as described earlier [25] with minor modifications. These cells were maintained at 37°C in DMEM with 10% fetal bovine serum. The chemoattractant consisted of COS-1 cell conditioned media containing the various ATX isoforms diluted in DMEM/0.1% BSA, fatty acid free (DB), with and without LPC (18:1) as indicated. Assays were incubated under standard conditions for 3.5 hr. Migration responses were assessed by counting nuclei that had traversed the pores and attached to the lower surface of the gelatin-coated membrane (for each chamber, six fields for each condition, at 100× magnification). Values were expressed as % of the response to WT-ATX in the absence of endogenous LPC.

### Enzyme Assays

Reaction buffer (TCB) was 50 mM Tris pH 7.4, 5 mM CaCl_2_, 1 mg/ml BSA (fatty acid free); substrates and their concentrations were included as indicated. LPC hydrolysis assays were based on detection of released choline. The reactions contained choline oxidase (0.1 U/ml), peroxidase (1.0 U/ml), and Amplex UltraRed (10 μM). Product formation kinetics (peroxide-driven Amplex UltraRed hydrolysis to yield fluorescent resorufin) was monitored by fluorimetry (Ex560 nm/Em590 nm). The assays for hydrolysis of pNpTMP [14] and FS-3 [22] were adapted from previously published methods. Reaction products were detected directly by optical absorbance (A405 nm for pNpTMP) or by fluorimetry (Ex494 nm/Em520 nm for FS-3). Reactions (50 μl in 96 well microplates) were initiated by addition of ATX (enzyme activity equivalent to 30 – 60 ng WT-ATX protein) and run at 37°C in a Tecan Genios Plus plate reader. Automated readings were performed at 15-minute intervals over a period of 2–3 hours. Slopes were determined for the linear (steady-state) portion of each reaction profile and used as reaction rates to calculate kinetic parameters. For the conditioned media experiments, background activity was measured using media from mock transfections and was subtracted from total activities to calculate ATX-specific activities. Regression analyses were calculated using Prism 4.0b (GraphPad Software, Inc, San Diego, CA).

## Abbreviations

ATX: (autotaxin); DB: (DMEM supplemented with 0.1% FAF-BSA); FAF-BSA: (fatty acid-free bovine serum albumin); LPA: (lysophosphatidic acid); LPC: (lysophosphatidylcholine); LPLD: (lysophospholipase D); NPP: (nucleotide pyrophosphatase and phosphodiesterase); PDE: (phosphodiesterase); pNpTMP: (p-nitrophenyl-TMP).

## Competing interests

The authors declare that they have no competing interests.

## Authors' contributions

EK performed the mutagenesis, construction of vectors, and ATX expression. RWB developed the enzyme assay methodology. DDR analyzed and interpreted data. TC performed the enzyme activity and migration assays. MLS and TC designed the study, performed statistical analysis, and wrote the manuscript. All authors read and approved the final manuscript.
